# Robust edge-based biomarker discovery improves prediction of breast cancer metastasis

**DOI:** 10.1186/s12859-020-03692-2

**Published:** 2020-09-30

**Authors:** Nahim Adnan, Chengwei Lei, Jianhua Ruan

**Affiliations:** 1 Department of Computer Science, The University of Texas at San Antonio, One UTSA Circle, San Antonio, 78249 TX USA; 2 Department of Computer & Electrical Engineering/Computer Science, California State University, Bakersfield, 9001 Stockdale Highway, Bakersfield, 93311 CA USA

**Keywords:** Biomarker discovery, Network-based classification, Breast cancer metastasis, Gene expression, Breast cancer prediction, Comparative analysis

## Abstract

**Background:**

The abundance of molecular profiling of breast cancer tissues entailed active research on molecular marker-based early diagnosis of metastasis. Recently there is a surging interest in combining gene expression with gene networks such as protein-protein interaction (PPI) network, gene co-expression (CE) network and pathway information to identify robust and accurate biomarkers for metastasis prediction, reflecting the common belief that cancer is a systems biology disease. However, controversy exists in the literature regarding whether network markers are indeed better features than genes alone for predicting as well as understanding metastasis. We believe much of the existing results may have been biased by the overly complicated prediction algorithms, unfair evaluation, and lack of rigorous statistics. In this study, we propose a simple approach to use network edges as features, based on two types of networks respectively, and compared their prediction power using three classification algorithms and rigorous statistical procedure on one of the largest datasets available. To detect biomarkers that are significant for the prediction and to compare the robustness of different feature types, we propose an unbiased and novel procedure to measure feature importance that eliminates the potential bias from factors such as different sample size, number of features, as well as class distribution.

**Results:**

Experimental results reveal that edge-based feature types consistently outperformed gene-based feature type in random forest and logistic regression models under all performance evaluation metrics, while the prediction accuracy of edge-based support vector machine (SVM) model was poorer, due to the larger number of edge features compared to gene features and the lack of feature selection in SVM model. Experimental results also show that edge features are much more robust than gene features and the top biomarkers from edge feature types are statistically more significantly enriched in the biological processes that are well known to be related to breast cancer metastasis.

**Conclusions:**

Overall, this study validates the utility of edge features as biomarkers but also highlights the importance of carefully designed experimental procedures in order to achieve statistically reliable comparison results.

## Background

Breast cancer has been identified as the prevalent diagnosed disease and also been the second most leading cause of death in western women [1]. Almost one woman out of 8 has the chance of being diagnosed with breast cancer over the course of their lifetime [2]. Spreading of cancer into the other organs within the body after the treatment/surgery of cancer is termed as metastasis and about 5% of women are affected by breast cancer metastasis [3]. Tumor size, histology, lymph node status and other factors have been considered for early diagnosis but later these aforementioned factors were found to be insufficient. Lately, molecular profiling of primary breast cancerous tissues has enabled the development of machine learning models for early prediction of metastasis. For prediction purpose, the patient being metastasis-free for at least 5 years and metastasis within 5 years are classified as good and poor outcomes respectively.

Prognostic gene biomarkers which includes the most differential genes between good and poor outcome were proposed for the diagnosis of breast cancer metastasis [1, 4–6]. Surprisingly, gene biomarkers varied across different studies, posing a significant challenge of identifying robust gene biomarkers [7]. Part of this dilemma can be attributed to the fact that multiple prognostic gene biomarkers may have similar and weak correlation to the disease [7, 8]. Biologically, proteins work together within a cell to accomplish a biological process and cancer is caused by the de-regulation of those biological processes. Motivated by the biological nature of proteins, some studies combined network information (PPI, co-expression network, metabolic network, pathway information) with the gene expression to acquire more accurate and robust network biomarkers [9–19]. These methods utilize gene modules (i.e., a set of genes as a network biomarker) as features which are more distinguishable between good and poor outcome exploiting the given network information and quantification of a gene module is done by averaging the expressions of the genes within the module. Not all gene modules are used as features in the prediction model; initial statistical significance test is conducted to obtain a smaller set of gene modules with higher differentiability between the good and poor outcome in some of the proposed methods [9, 13]. In other studies, edge (i.e., gene pair) biomarkers were used for better prediction accuracy. In these methods, differential edge biomarkers are used to capture the rewired interactions using co-expression network [20] and PPI [21] as the background network. Another approach PhenoNet [22] was proposed to capture differential regulation in PPI where pathway information is used as the baseline. Some methods combining differential expression and gene regulatory networks have not only achieved better prediction accuracy but also helped identify biomarkers that are well associated with the studied disease [23, 24]. However, some doubt still exist whether network biomarkers offer better prediction accuracy and robustness compared to gene expression alone [25, 26].

Each of the above-mentioned methods utilizes machine learning prediction model for the discovery of the biomarkers. Different prediction models are found to be performing better in different studies. For instance, Linear Regression, Logistic Regression (LR), Random Forest (RF) and Linear SVM (SVM) are the models mostly used to predict the disease in question. In biomarker discovery, the interpretability of the trained model is very crucial to identify the genes responsible for the cause of the disease. Random forest is very easy to interpret and it also provides a feature importance measurement which can be easily used to identify potential biomarkers. Another advantage of random forest is that it can be used as a feature selection tool to acquire the most responsible disease-associated genes as well as used as an accurate prediction model [27, 28].

In this current study, we present two edge-based feature types based on PPI and CE networks and tested on the Amsterdam Classification Evaluation Suite (ACES) dataset which includes more than 1600 patients from twelve patient cohorts. Comparative analysis is done on the edge-based feature types compared to gene-based feature type from several different aspects. Prediction accuracy of the feature types is evaluated for multiple prediction models (i.e., RF, SVM, and LR models). Multiple evaluation metrics are considered to validate which feature type is more accurate. All of the comparisons are done considering with/without the statistical significance. In addition, a novel procedure has been proposed to evaluate the robustness of different feature types using the RF model and to identify the most significant biomarkers for each of the feature types. Results reveal that edge-based features provide not only more accurate prediction than gene-based features under multiple performance metrics but also much more robust performance than the latter. The top biomarkers from edge-based feature types are statistically more significantly enriched in the biological processes that are well known to be related to breast cancer metastasis.

## Results and discussions

### Evaluation of prediction performance using random forest

**Fig. 1:**
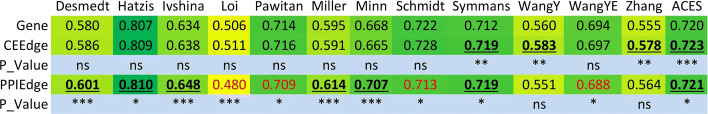
AUC evaluation of edge-based RF model compared to gene-based RF model. The highlighted entry as “Bold” and “Underlined” indicates that the edge-based model outperformed gene-based model with statistical significance. Significance of the *p*-value of the paired t-test between the edge- and gene-based model AUCs is provided in the “P _Value” row under that edge-based feature type row. ***, ** and * indicate that the *p*-value is lower than 0.0005, 0.005 and 0.05 respectively. “ns” indicates that the *p*-value is not statistically significant. “Red” entry indicates that gene-based model outperformed edge-based model with statistical significance. Colors are provided for better visualization of the results

Thirteen (13) datasets (i.e., 12 separate studies and combined 12 studies as one dataset) were used to evaluate the performance of prediction models. AUC comparison of RF model is shown in Fig. 1. Overall it can be observed that both CEEdge and PPIEdge feature types perform better than gene-based feature type, while some of the difference lack statistical significance. Very low AUC scores of Loi, WangY and Zhang datasets suggest that the model predictions are almost like random prediction. This is most likely due to the small dataset size and/or imbalanced class distribution of metastatic and non-metastatic patients in those datasets (see Table 8).

**Fig. 2:**
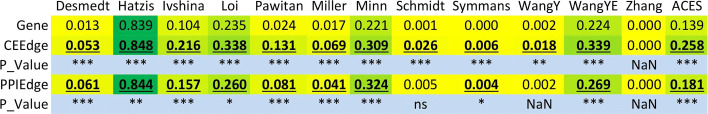
F1-score evaluation of edge-based RF model compared to gene-based RF model, with default class probability threshold. The explanation of the highlighted entry in figure is similar as in Fig. 1

**Fig. 3:**
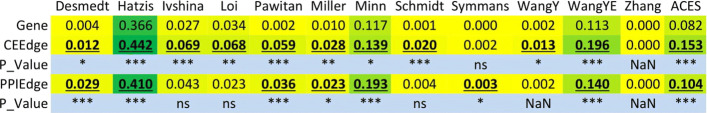
Kappa evaluation of edge-based RF model compared to gene-based RF model, with default class probability threshold. The explanation of the highlighted entry in figure is similar as in Fig. 1

For a better understanding of the prediction accuracy of different feature types, F1- and Kappa-scores were also evaluated, which relied on specific class probability thresholds. The first computation of both metrics was based on the class prediction by the RF using the default probability threshold (0.5). The results for F1-score and Kappa are given in Figs. 2 and 3 respectively. While the edge-based models are consistently better than the gene-based model, it is evident that the values for both F1-score and Kappa in most models are fairly low, except the Hatzis, Minn and WangYE datasets. As many of the datasets are highly imbalanced (with much more negative samples), the predicted probabilities returned by the RF model mostly contained probabilities much lower than the 0.5 threshold which resulted in very few positive predictions. A few NaN values for the *p*-value of paired t-test were observed for the F1-score and Kappa comparison due to the values being zero in all repetitions (i.e., all samples classified as negative).

**Fig. 4:**
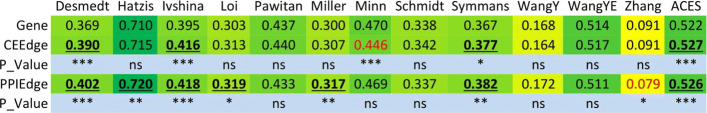
F1-score evaluation of edge-based RF model compared to gene-based RF model, with optimal class probability threshold. The explanation of the highlighted entry in figure is similar as in Fig. 1

**Fig. 5:**
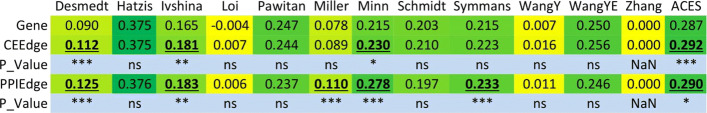
Kappa evaluation of edge-based RF model compared to gene-based RF model, with optimal class probability threshold. The explanation of the highlighted entry in figure is similar as in Fig. 1

Observing the problem of using the default probability threshold on producing meaningful F1-score and Kappa for evaluation purposes, a different strategy was employed to predict the class labels using the “optimal” probability threshold determined from the ROC curve (for details see 2). The results for the F1-score and Kappa using the optimal probability threshold are shown in Figs. 4 and 5 respectively. While the Kappa scores are still very low in Loi, Miller, WangY and Zhang datasets due to their small dataset sizes and/or skewed class distribution, the overall evaluation results based on F1-score and Kappa are much stable than the results found using the default probability threshold of the RF model. Based on this evaluation strategy, it is again evident that the edge-based models consistently outperformed gene-based model with/without statistical significance.

The overall performance comparison of RF models using different metrics is summarized in Table 1. Based on AUC measurement, CEEdge outperforms gene-based feature in 4 datasets with statistical significance and also outperforms in another 7 datasets albeit with no statistical significance; PPIEdge outperforms gene-based feature in 7 datasets whereas it is outperformed by gene-based feature in 4 datasets. Using F1-score and Kappa, it is clear that CEEdge and PPIEdge performed much better than the gene-based model. With the default class probability threshold, CEEdge and PPIEdge outperform gene-based model in 12 and 10 datasets respectively for F1-score and in 11 and 8 datasets respectively for Kappa. When the optimal class probability threshold is used, the comparison results are very similar, with CEEdge winning F1-score in 10 datasets and Kappa in 10 datasets, and PPIEdge winning F1-score in 8 datasets and Kappa in 9 datasets with/without statistical significance.

**Table 1:** Performance summary of CEEdge and PPIEdge feature types compared to gene-based feature type in RF models. “Win” indicates that the edge-based feature type outperformed gene-based feature type and “Lose” indicates that the gene-based feature type outperformed edge-based one. “SS” and “NSS” indicate that win/lose was statistically significant and not statistically significant respectively

Evaluation Metric Type	Feature	Win	Lose	Win	Lose	Equal
	Type	SS	SS	NSS	NSS	/NaN
AUC	CEEDge	4	0	7	2	0
	PPIEdge	7	4	1	1	0
F1-score (0.5 probability threshold)	CEEdge	12	0	0	0	1
	PPIEdge	10	0	1	0	2
Kappa (0.5 probability threshold)	CEEdge	11	0	1	0	1
	PPIEdge	8	0	2	1	2
F1-score (Optimal probability threshold)	CEEdge	4	1	6	2	0
	PPIEdge	7	1	1	4	0
Kappa (Optimal probability threshold)	CEEdge	4	0	6	2	1
	PPIEdge	6	0	3	3	1

### Performance evaluation using support vector machines and logistic regression

**Fig. 6:**
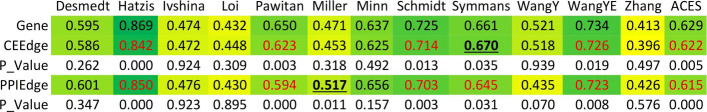
AUC comparison of edge-based and gene-based SVM models. The explanation of the highlighted entry in figure is similar as in Fig. 1

**Fig. 7:**
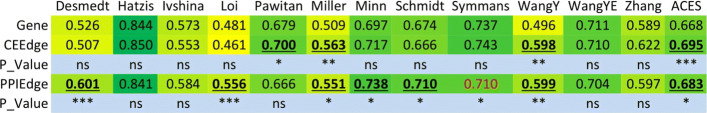
AUC comparison of edge-based and gene-based LR models. The explanation of the highlighted entry in figure is similar as in Fig. 1

AUC comparison for SVM and LR models between the gene- and edge-based feature types are given in Figs. 6 and 7 respectively. From the results of the SVM model, it is evident that SVM has very poor performance in most of the smaller datasets such as Ivshina, Loi, Miller, WangY, and Zhang. The AUC results are worse than the random prediction in 4 of the 13 datasets. A similar trend can also be observed from the result of the LR model. The overall performance comparison of SVM and LR models using gene- and edge-based feature types is provided in Table 2. Gene-based feature type outperforms edge-based feature types in most of the datasets (with or without statistical significance) for SVM model. The poor performance of edge-based feature types in the SVM model is due to a large number of edge features compared to gene features. The number of edge-based features is at least 13 times larger than the number of gene features. Moreover, gene expression data contains a lot of noisy features. When edge features were generated it contained much more noisy features than the gene-based features. Another problem is that SVM does not have any built-in feature selection method. A single SVM model built with these huge number of edge features is unable to obtain a good decision boundary between metastatic and non-metastatic class. On the other hand, CEEdge and PPIEdge feature type outperform gene-based feature type in 8 and 9 datasets respectively with/without statistical significance in LR-based models. The good performance of edge-based LR models is due to the L1 penalization in the model building process which provides sparse coefficients for edge features and ensures only the discriminative edge features contributed to the final model. Additionally, the optimized C parameter by inner cross-validation also leads to good LR models for edge-based feature types.

**Table 2:** Performance summary of CEEdge and PPIEdge features compared to gene feature in SVM and LR models. Table description is similar to as in Table 1

Prediction Model	Feature Type	Win SS	Lose SS	Win NSS	Lose NSS	Equal
SVM	CEEdge	1	5	1	6	0
	PPIEdge	1	6	4	2	0
LR	CEEdge	4	0	4	5	0
	PPIEdge	7	1	2	3	0

The fact that the edge-based feature types perform significantly better than the gene-based feature type in most of the analyzed datasets from multiple evaluation metrics suggests a bona fide benefit of the network information in better classifying metastatic and non-metastatic patients. It is worth noting that the number of edges was much larger than the number of genes in the analysis, which underlines the effectiveness of RF and LR’s implicit feature selection. It also suggests that edge-based features possess more discriminative power than gene-based features albeit a huge number of features. On the other hand, this indicates that a significant amount of feature redundancy may be present in the edge-based datasets, which necessitates a detailed feature robustness analysis.

### Performance comparison with existing methods

To further evaluate the discriminating power of edge-based features, the PPIEdge feature type was used to construct multiple random forest classifiers and compared with two state-of-the-art network-based methods. Park [10] produces genesets (group of genes) using the linkage algorithm and uses the average of the genes belonging to a geneset as the composite feature. In Feral [18], a geneset is created by taking it’s 9 neighbors randomly for each gene. A feature group is created by applying the operators (mean, maximum, minimum, median, variance) on the geneset for each gene. Sparse group lasso is used as the prediction model.

**Fig. 8:**
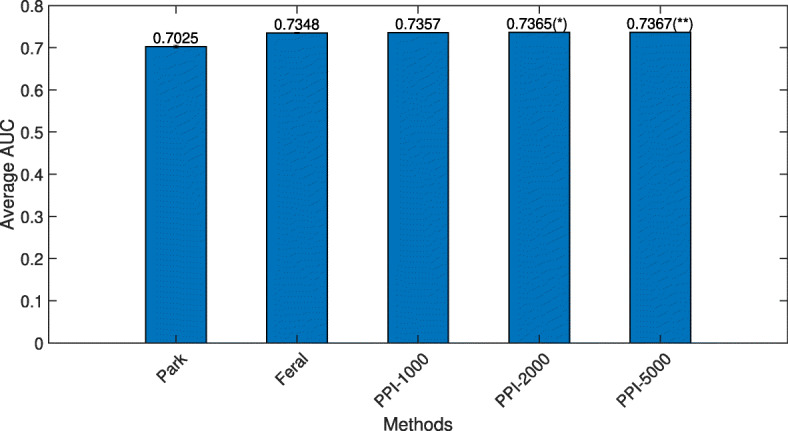
AUC comparison of PPIEdge feature type compared to Park and Feral. Error bar denotes the 95% confidence interval. “PPI-1000” denotes RF model with 1000 decision trees (estimators) trained on PPIEdge feature type. Value on the top of the bar indicates the average AUC of that method. “*” indicates the significance of the *p*-value obtained from the paired t-test between the AUCs of Feral and PPI methods. “*” and “**” indicate that the *p*-value is lower than 0.05 and 0.005 respectively

Figure 8 shows the average AUC of different methods. Feral and PPIEdge methods clearly outperform the Park method significantly whereas the AUCs of PPIEdge-based classifiers (with the number of estimators range from 1000 to 5000) are slightly better than Feral. Note that Feral (and many other existing methods for network-based classifiers) is significantly more complex than our method. Feral used multiple group operators as well as random neighbor selection to create a large number of feature groups, and a classification algorithm tailored towards the feature types (sparse group lasso). It is unknown whether the performance gain of Feral came primarily from the network-based features, or the lasso-based regularization, or a combination of both. Performance evaluation of complex models is often inflated, as the testing data may have played a role in the construction of the “optimal” evaluation pipeline (explicitly or implicitly). In contrast to Feral, our edge-based classifier used a very simple feature extraction method (pairs of genes defined by a given network) and common underlying classification algorithms for both gene-based and network-based classification. Therefore, the evaluation results are less likely to be biased, and clearly show that the performance improvement originated from the features.

### Running time and memory requirement

To get an overview of the running time and memory requirement of the different machine learning models, the running time and peak memory consumption for executing a single fold of ACES dataset was evaluated and provided in Tables 3 and 4 respectively. RF model seems to be the most efficient in both running time and memory consumption. Although the LR model requires a lot of memory the execution time is much lower compared to the SVM model. Overall, it is evident that the running time and memory consumption CEEDge and PPIEdge consumed a lot of memory compared to the gene feature type because of the huge number of features in edge feature types. Overall, the running time of SVM model is extremely poor compared to the RF and LR models.

**Table 3:** Runtime evaluation (in seconds) of RF, SVM and LR models for different feature types

Model	Gene	CEEdge	PPIEdge
RF	12.2	43.5	46
SVM	126	1513.6	1614.5
LR	11.3	140.6	144.1

**Table 4:** Peak memory consumption evaluation (in MegaByte (MB)) of RF, SVM and LR models for different feature types

Model	Gene	CEEdge	PPIEdge
RF	687.1	6162.8	6743.4
SVM	531.8	6129.4	6737.4
LR	611.5	7209.1	8140.7

### Robustness evaluation

**Fig. 9:**
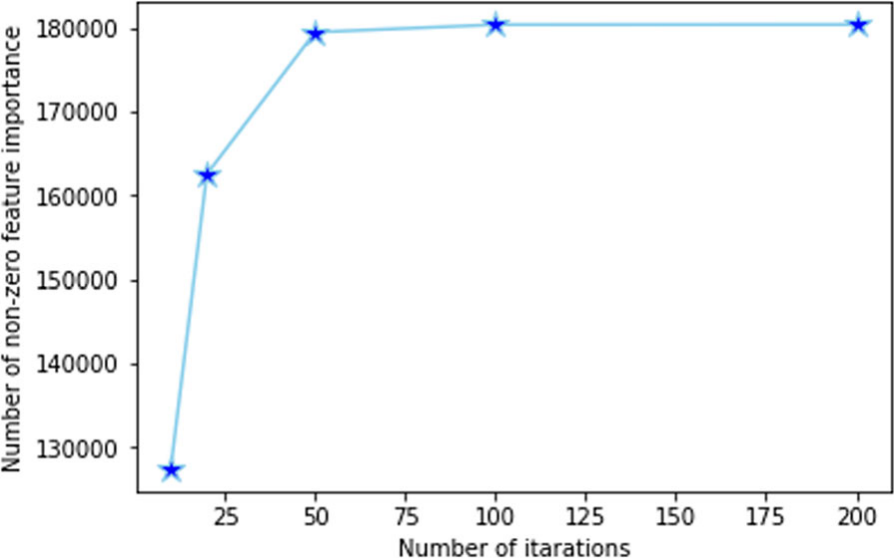
Number of non-zero feature importance values after running RF for various number of iterations for PPIEdge

For robustness analysis, RF model was used as the base model. A single RF model could not provide feature importance values for all of the features in a feature type. Thus, we employed an approach to building RF model for multiple iterations (with different seeds to generate random numbers) on the same input dataset can increase the features sampled and produce feature importance values for more features. We did an experiment to determine the number of features having non-zero feature importance value with respect to the number of iterations of RFs. RF with 500 estimators (decision trees) was trained for 10, 20, 50, 100, 200 iterations on PPIEdge feature type. Figure 9 shows the impact of building RF model for multiple iterations. It is evident that the chance of being sampled in the random subset of features in the node splitting of the decision tree increased as the number of iteration increased. All of the features had a feature importance value after 200 iterations. The approach was employed to give each feature a chance of being in the top feature list. Another advantage of our approach is that the bootstrapping process added a statistical significance measure for the feature importance scores.

ACES dataset as a whole was used for the evaluation of feature robustness in our analysis. Gene stability among the separate datasets was measured as the robustness in previous studies [18]. Some problems such as uneven patient samples in different studies, class imbalance problem, uneven distribution of breast cancer sub-types have a strong impact on the stability of the founded gene signatures (data not shown). To circumvent this problem, in this study, the ACES dataset was partitioned into two equal-sized sub-samples with similar class and subtype distributions.

**Fig. 10:**
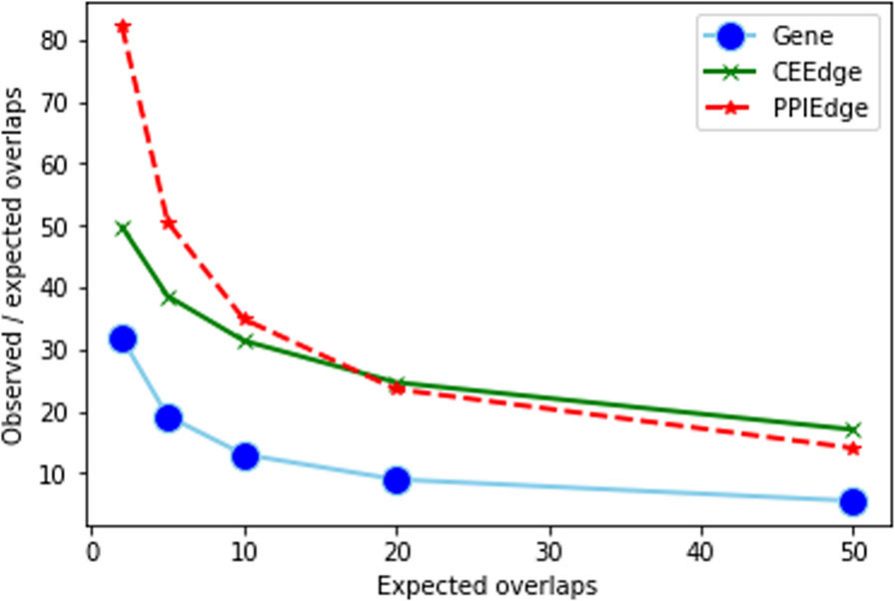
Robustness measure (y-axis) for Gene, CEEdge and PPIEdge features at different levels of expected overlaps (x-axis)

Figure 10 shows the ratio of observed to the expected number of overlaps at different levels of expectations. (The number of top features needed at different expectation levels for each feature type is provided in Table 5). It was clearly observable that CEEdge and PPIEdge feature types had much higher overlap ratios compared to gene-based feature type. This finding indicates that network integration with gene expression provided more robust features than the gene expression alone, which is in conformity with a previous study [18], contrary to two other studies [26, 29]. This discrepancy may be attributed to the external feature selection for the network-based models where no external feature selection was employed in our evaluation. The mean difference in the overlap ratios is also statistically significant (see Table 6). The ratio decreases as the expectation level increases, which is expected because the significance of the selected features decreases as the number of selected features increases, resulting in more noise in the selected features.

**Table 5:** The number of top features selected for different feature types with various expected number of overlaps in feature robustness analysis

Expected overlaps	2	5	10	20	50
Gene	159	252	357	504	798
CEEdge	565	895	1268	1791	2836
PPIEdge	598	947	1342	1895	3001

**Table 6:** *p*-values of the paired t-test between the robustness of gene- and edge-based feature types for different expected overlaps

Expected overlaps	2	5	10	20	50
CEEdge *p*-value	5.3E-08	2.5E-11	2.4E-13	2.2E-15	1.8E-17
PPIEdge *p*-value	2.7E-16	7.1E-17	1.5E-17	8.3E-18	3.7E-19

### Gene ontology enrichment evaluation

To identify the biological functions of the top significant biomarkers (details of the process of finding top biomarkers, see “Methods” section) for different feature types, Gene Ontology analysis was done with the top 500 genes for each feature type and the results are provided in Table 7. Cell cycle and cell cycle-related processes are in the top enriched biological processes for all feature types, which confirms that the procedure of feature ranking is complete. Other GO terms including antigen processing, cell-cell adhesion, telomere maintenance, natural killer cell mediated cytotoxicity and focal adhesion, which are well-known biological processes involved in metastasis are found to be significant in some but not all feature types. Some other important signaling pathways such as T-cell receptor, p53, Wnt, Jak-stat are also found in different feature types. Note that the *p*-values of the enrichment score was adjusted for multiple hypothesis testing. From the table, it is evident that PPIEdge has the most significant *p*-values for most of the GO terms which indicates that PPIEdge is more biologically relevant than CEEdge feature type, and potentially can provide important biological information for a better mechanistic understanding of the development of metastasis. Moreover, our approach for top gene identification is much robust because almost the same biological GO terms are found in all the three feature types.

**Table 7:** Gene Ontology(GO) analysis of different feature types. The entries for Gene, CEEdge and PPIEdge indicate the Benjamini corrected *p*-value for that GO term

GO term	Gene	CEEdge	PPIEdge
Cell cycle	2.3E-15	4.5E-19	2.0E-30
Sister chromatid cohesion	4.4E-5	1.7E-5	5.1E-12
Anaphase-promoting complex-dependent catabolic process	4.4E-5	8.8E-6	1.4E-7
Negative regulation of ubiquitin-protein ligase activity involved in mitotic cell cycle	3.9E-4	3.2E-4	1.1E-5
Antigen processing and presentation of exogenous peptide antigen via MHC class II	7.1E-4	3.8E-2	2.9E-3
DNA repair	4.2E-1	3.8E-1	8.8E-3
Positive regulation of telomere maintenance via telomerase	3.6E-1		1.4E-2
Cell-cell adhesion			1.7E-2
T cell receptor signaling pathway	6.0E-1	9.7E-1	6.7E-2
p53 signaling pathway	4.9E-2	1.1E-1	6.4E-2
RB Tumor Suppressor/Checkpoint Signaling in response to DNA damage	7.6E-1	9.0E-1	1.2E-1
Wnt signaling pathway, planar cell polarity pathway	8.1E-1	5.3E-1	2.1E-1
Natural killer cell mediated cytotoxicity	9.5E-1		8.8E-1
Focal adhesion			9.7E-1
Small cell lung cancer	7.0E-1	6.3E-1	3.8E-2
Jak-STAT signaling pathway	9.7E-1	9.9E-1	9.5E-1
Tumor necrosis factor-mediated signaling pathway	6.5E-1	5.9E-1	2.1E-2

**Table 8:** Specification of the studies in ACES

Dataset	Geo accession no.	No. of poor	No. of good	Total patient
Desmedt	7390	56	127	183
Hatzis	25066	102	48	150
Ivshina	4922	30	72	102
Loi	6532	24	33	57
Pawitan	1456	33	114	147
Miller	3494	21	68	89
Minn	2603	21	44	65
Schmidt	11121	24	145	169
Symmans	17705	37	187	224
WangY	5327	10	42	52
WangYE	2034	88	169	257
Zhang	12093	9	112	121
ACES		455	1161	1616

## Conclusions

In this study, we propose two types of edge-based features based on protein-protein interaction network and gene co-expression network, and compare them with the gene-based features under different evaluation contexts. Edge-based random forest (RF) and logistic regression (LR) models outperformed corresponding gene-based models significantly in predicting breast cancer metastasis for multiple evaluation metrics. The improved accuracy of RF and LR models can be partially attributed to the built-in feature selection mechanism which utilizes the most discriminative edge features into the model. In contrast, the poor performance of edge features in support vector machine (SVM) model is due to the much higher dimension of the edge-based features and the lack of internal feature selection in SVM model. A novel statistical procedure is proposed for robustness measure which is unaffected by the small sample size, class imbalance problem and the uneven distribution of breast cancer sub-types within the sub-samples used for robustness analysis. Although edge-based feature types are much larger in feature dimension than the gene-based feature type, the experimental results reveal that edge-based features are consistently much more robust than the gene-based features at different levels of statistical significance. We also introduce a rigorous RF-based strategy for selecting top-ranked genes as biomarkers for each of the feature types. Top biomarkers are found to be significantly enriched in breast cancer metastasis-related biological processes not only for edge-based feature types but also for the gene-based feature type, which clearly demonstrated the robustness and accuracy of the proposed approach in identifying biomarkers responsible for metastasis. Finally, we conclude that the edge biomarkers are more robust and provide better prediction accuracy than gene expression alone, signifying the potential of edge-based biomarker discovery for the interpretation of the underlying mechanism of the breast cancer metastasis.

## Methods

### Gene expression and network data

**Gene expression dataset.** The gene expression dataset for breast cancer metastasis was collected from the Amsterdam Classification Evaluation Suite (ACES) [25]. ACES is a compilation of twelve (12) separate breast cancer studies from NCBI’s Gene Expression Omnibus. The ACES dataset used only the 133A platform. It discarded the same GEO patient IDs from multiple studies. Using R’s *arrayQualityMetrics*, array quality control was checked for all the patient samples belonging to the same study. RLE (Relative Log Expression) or NUSE (Normalized Unscaled Standard Error Plot) analysis were employed to exclude the patient sample outliers from further examination. After executing all these preprocessing steps, 1616 patient samples were selected in the final patient cohort. The expression arrays of these 1616 patients were normalized altogether using the *justRMA* method from *R*. Probe intensities were log-normalized and mean centered for each sample. Finally, 12750 gene probes remained in ACES dataset. Additionally, there were batch effects in the dataset due to the collection of expression data from different location and for different sub-types of breast cancer. R’s *combat* was employed to remove the batch effects from the dataset. For details of the dataset and preprocessing steps, see the article [25].

The class label of a patient is determined as negative (good) class if the patient was free from recurrence of cancer for at least five (5) years. If cancer occurred again within five (5) years for a patient, the patient is classified as a positive (poor) class. The detailed information about the studies and the class distributions are provided in Table 8.

**Protein-protein interaction network (PPI).** The protein-protein interaction network (PPI) was curated from the BioGrid (version 3.4.149) interaction database [30]. The network only considered the edges if two genes were in the 12750 genes of ACES dataset. After discarding self-edges, 180371 edges for 12750 nodes (i.e., genes) remained in the PPI network.

**Gene co-expression network (CE).** A global gene co-expression network was used as another network in the evaluation. Pearson correlation coefficients [31] between the genes’ expressions in the ACES dataset was computed. If two corresponding genes were present within the top-*k* most co-expressed genes from each other, an edge was created in the network. To keep the network structure (i.e., number of edges and degree distribution) like the PPI network, the number of neighbors (*k*) was set to 84. The co-expression network contained 161042 edges for 12750 nodes (i.e., genes) and the power-law distribution was observed for the degree distribution of the network; it is the typical distribution of the real-world network [32].

**Fig. 11:**
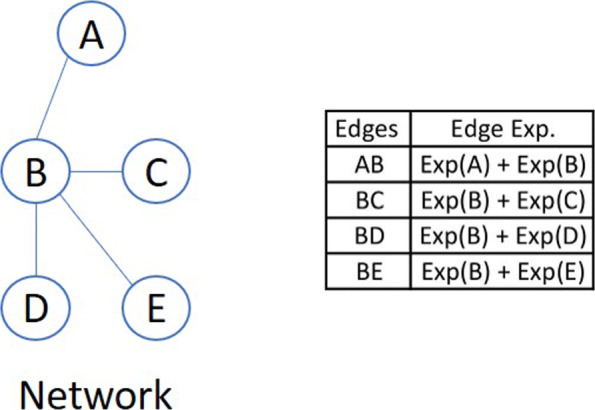
Edge-based feature from a given network

**Edge-based feature type.** The edge-based feature type utilizes all the edges belonging to a given network (i.e., PPI and CE network). Expression of an edge was calculated as the sum of the expression of the two genes corresponding to that edge (Fig. 11). The edge-based feature type from PPI and CE network are termed as “PPIEdge” and “CEEdge” respectively.

### Prediction performance comparison

To measure the prediction performance of different feature types, an RF model was built on each of the feature types for the 13 datasets separately. An RF model with 100 estimators (i.e., 100 decision trees) was used with other default settings from Sklearn API [33] in python. The performance was measured using 5-fold cross validation for 100 repetitions. Three types of metrics were used to evaluate the prediction performance of different feature types. These are the area under the receiver-operating characteristics curve (AUC) [34], the Cohen’s Kappa [35] and the F1-score [36]. AUC score is dependent on the predicted probability vector for test instances by the prediction model (raw predicted probabilities obtained from RF model). However, Kappa and F1-score are computed by the class prediction of test instances rather than the predicted probabilities from the prediction model. For evaluating Kappa and F1-score, two strategies were employed. RF’s default class prediction was used to compute the metrics in one strategy. Another strategy was to make a prediction of classes (i.e., two class prediction) using the optimal probability threshold. For this experiment, 200 repetitions of 10-fold cross validation were executed to evaluate the Kappa and F1-score. Each fold was considered as a test set and 80% and 20% (randomly chosen) of the remaining 9 folds were divided into training and validation sets respectively. RF model was trained on the training set and optimal probability threshold was chosen using the predicted probabilities returned by the trained RF model for the validation set instances. Optimal probability threshold was determined as the shortest distance from the top-left corner to the ROC curve as defined in the Eq. 1 [37].

1 $$  distance = \sqrt{(1 - sensitivity)^{2} + (1 - specificity)^{2}}  $$

Finally, predicted probabilities were obtained for the test set instances from the trained RF model and the class labels were decided using the optimal probability threshold obtained from the validation set. The negative class was set for the test instance whose probability was lower than the optimal probability threshold; it was set to positive class otherwise. Kappa and F1-score were recomputed to evaluate the prediction performance of the feature type based on the second strategy. In this procedure, no bias/overfitting was introduced into the computation of Kappa and F1-score because optimal probability threshold was determined by completely separate validation set rather than utilizing training or test sets [38].

Linear kernel-based Support Vector Machine (SVM) and Logistic Regression (LR) were also employed to evaluate the prediction accuracy of the different feature types. AUC scores of 5-fold cross validation for 10 repetitions were measured for the comparison. Default settings of Linear SVM from Sklearn API [33] in python were used. For LR model, inverse of regularization strength C parameter was optimized by an inner 5-fold cross validation on the training set. C parameter was searched in the set {0.001, 0.01, 0.1, 1, 10, 100}. Additionally, L1 penalty was set in the LR model to provide sparse feature coefficients. Finally, LR model was trained on the training set with the best C parameter found by the inner cross validation.

Paired t-test between gene- and edge-based feature type was done to provide statistical significance of the comparisons. A *p*-value was computed for the comparison of the mean of the feature types for each of the evaluation metrics. The statistical significance threshold was set to 0.05 for each comparison. The comparative result was provided for both statistical significance and insignificance.

Moreover, to evaluate the significance of the edge-based features (gene pairs), an RF model trained on PPIEdge feature type was compared to two existing network-based methods, Park [10] and Feral [18]. RF model with 1000, 2000, and 5000 estimators (decision trees) was trained on the PPIEdge feature type to provide optimal predictive accuracy. 10-fold cross-validation was repeated for 30 times for this comparison.

### Robustness analysis

To measure the robustness of the feature types, we relied on the feature importance scores returned by the RF model. The robustness of a feature type is measured by first drawing random samples from the dataset and identify *x* top-ranked features by importance score from each sampled dataset, and then calculate the ratio between the observed number of common features identified from the two subsets of samples, and the expected number of common features by chance. However, a few important details are worth noting, in order to achieve statistically valid comparison results.

First, it is important to note that, because of the nature of its algorithm, RF model calculates the importance score only for a randomly sampled subset of features, and a zero value in the importance score simply means the feature was never sampled in the model building process. While the number of estimators (decision trees) needed to achieve a good prediction performance is usually small (≈100 in our case) and remains similar for gene-based and edge-based features, the number of estimators needed for identifying top-ranked features and performing robustness analysis is much larger, as the ranking is meaningful only when every feature has been sampled (and scored) at least once. Also note that more estimators are needed for edge features than gene features to achieve similar coverage, due to the large number of edge features.

Another important issue is that in order to compare the robustness between edge features and gene features, the number of selected top-features needs to be adjusted for different feature types according to the number of features available. This is to ensure that the same fold enrichment of observed/expected number of common features from different feature types should correspond to similar statistical significance. As an illustration, assume that we have 10,000 genes and 160,000 edges. Say that we choose top 200 genes from two independent sets of patients, we would expect 4 =(200*200/10,000) genes in common between the two gene lists by chance. If there were actually 16 genes in common, the level of fold enrichment is 16 / 4 = 4, and the *p*-value is 2.4e-6 (fisher’s exact test based on hypergeometric distribution). To compare the robustness between gene-based features and edge-based features, say that we also choose top 200 edge-based features from each dataset. The expected overlap between the two sets of edges is 200 * 200 / 160,000 = 0.25. An actual overlap of one edge would represent a 4-fold enrichment as in gene-based features, but is statistically insignificant (p = 0.22). On the other hand, if we choose proportionally (i.e., 200 x 160,000 / 10,000 = 3,200 edges), we would expect 3,200 * 3,200 / 160,000 = 64 common edges by chance, and an actual overlap of 256 edges would represent the same 4-fold enrichment, but is statistically much more significant than the gene-based features (*p*-value = 3.4e-78 for edges vs *p*-value = 2.4e-6 for genes). In comparison, if we choose 800 edges from each dataset, the expected number of common edges is 4 (same as in the case of gene features), and an actual overlap of 16 edges will represent a 4-fold enrichment, with a statistical significance of 4.1e-6, which is very comparable to the case of gene-based features.

With the above intuition, we decided to choose different number of features for gene-based and edge-based features separately, while keeping the expected number of overlaps the same for different types of features. Since the expected number of overlaps between two lists of features is calculated as *expected*_*#*_*of*_*overlaps*=(*#*_*of*_*selected*_*top*_*features*)^2^/(*total*_*#*_*of*_*features*), the number of selected top features is determined by:

2 $$ X = \sqrt{total\_\#\_of\_features \times expected\_\#\_of\_overlaps}  $$

Finally, to achieve an unbiased evaluation of robustness, the ACES dataset was randomly partitioned into two disjoint sub-samples, where each sub-sample had the same number of patients and similar metastatic/non-metastatic patient ratio. Top features were picked for a sub-sample from the returned feature importance vector for a feature type after training the RF model by that sub-sample. Multiple RF models were trained with different random states (i.e., random seed) on the same sub-sample to produce feature importance values for each feature belonging to the sub-sample. The number of estimators (decision trees) was chosen to be 300, 500 and 500 and the number of training iterations was assigned as 50, 200 and 200 for Gene, CEEdge and PPIEdge feature types respectively. Final feature importance of a feature was determined as the average of the non-zero values returned by the multiple trained RF models. Feature importance vector for each sub-sample was computed and top features were selected based on the highest feature importance values. Top *X* genes were selected from the two corresponding sub-samples and the robustness was measured as the ratio between the observed number of overlaps to the expected number of overlaps between the top *X* features of the two sub-samples. The process of splitting ACES into two sub-samples was repeated for 20 times so that a vector of 20 overlap ratios were obtained for each feature type for a specific expectation value (expected number of overlaps).

### Gene ontology enrichment analysis

Top genes were selected after conducting bootstrapping on 65% of the sub-sampled data (with replacement) of ACES for 1000 repetitions for each of the feature types. The number of estimators (i.e., decision trees) for gene, CEEdge, and PPIEdge feature types were kept at 1000, 12630, and 14123 respectively. The number of estimators in RF for CEEdge and PPIEdge was increased linearly according to the number of features in the gene-based RF model. For instance, the number of estimators for CEEdge was set to (1,000*161,042)/12,750 = 12630, where 1000 is the number of estimators in gene-based RF model, and 12750 (161042) the number of gene (CEEdge) features. Increasing the number of estimators in CEEdge and PPIEdge-based RF models provided a similar percentage of non-zero feature importance measures for CEEdge- and PPIEdge-based models compared to gene-based model. The final importance score of a feature (gene or edge) was computed as the average of the non-zero feature importance values from the feature importance values obtained from 1000 repetitions of RF (Recall that a zero value in feature importance from RF model simply means the feature was not sampled in the model building process). Sorting the final feature importance values in descending order for each feature type resulted in the feature ranking vector. Top 500 genes were selected from the sorted feature ranking vector for gene ontology enrichment analysis of the gene-based features. Top 500 genes for edge feature type were selected by pooling genes from the top-ranked edges until 500 genes were obtained. David Bioinformatics Resources 6.8 [39] web application was used for gene ontology enrichment analysis.

## Data Availability

The datasets used and/or analysed during the current study are available from the corresponding research articles cited.

## Abbreviations

ACES: Amsterdam classification evaluation suite
AUC: Area under curve
CE: Gene co-expression network
CEEdge: Edge-based features from CE network
GO: Gene ontology
Kappa: Cohen’s kappa statistic
Lose SS: Lose with statistical significance
Lose NSS: Lose without statistical significance
LR: Logistic regression model
MB: Megabyte
PPI: Protein-protein interaction network
PPIEdge: Edge-based features from PPI network
RF: Random forest model
SVM: Linear kernel-based support vector machine model
Win SS: Win with statistical significance
Win NSS: Win without statistical significance
